# Collaborative decision support and documentation in chemical safety with KnowSEC

**DOI:** 10.1186/s13321-016-0132-8

**Published:** 2016-04-23

**Authors:** Joachim Baumeister, Albrecht Striffler, Marc Brandt, Michael Neumann

**Affiliations:** Institute of Computer Science, University of Würzburg, Am Hubland, 97074 Würzburg, Germany; denkbares GmbH, Friedrich-Bergius-Ring 15, 97076 Würzburg, Germany; Section IV 2.3 Chemicals, The Federal Environment Agency (Umweltbundesamt), Wörlitzer Platz 1, 06844 Dessau-Roßlau, Germany

**Keywords:** Decision support, Knowledge-based systems, Ontologies, Expert systems, Semantic technologies

## Abstract

To protect the health of human and environment, the European Union implemented the REACH regulation for chemical substances. REACH is an acronym for Registration, Evaluation, Authorization, and Restriction of Chemicals. Under REACH, the authorities have the task of assessing chemical substances, especially those that might pose a risk to human health or environment. The work under REACH is scientifically, technically and procedurally a complex and knowledge-intensive task that is jointly performed by the European Chemicals Agency and member state authorities in Europe. The assessment of substances under REACH conducted in the German Environment Agency is supported by the knowledge-based system KnowSEC, which is used for the screening, documentation, and decision support when working on chemical substances. The software KnowSEC integrates advanced semantic technologies and strong problem solving methods. It allows for the collaborative work on substances in the context of the European REACH regulation. We discuss the applied methods and process models and we report on experiences with the implementation and use of the system.

## Background

In the year 2007 the European Union implemented the REACH regulation for chemical substances in order to protect the health of human and environment. The REACH regulation introduced the following processes for substances:

1. *Registration* Companies have to register all chemical substances they produce or import. In these registrations properties of the substance, the planned uses, and an assessment of hazards and potential risks and further information have to be documented.
2. *Evaluation* The European Chemicals Agency (ECHA) together with the member state authorities evaluates the registered substances and assess their potential risk to human health and environment.
3. *Authorization and restriction* A main task of the actual regulation of those chemicals posing a risk is to *efficiently* identify those substances that have properties making them “substances of very high concern” (SVHC). These substances are to be substituted in the long run. Also there is the possibility to place restriction on the use of chemicals that constitute a risk.

At the moment, about 13,400 substances are registered under REACH and it is expected that until 2018 this number will rise to at least 30,000. The high number of substances enforces the need for the authorities to employ effective techniques and processes for the assessment of these substances within the different procedures of the REACH regulation. Depending on the individual regulatory procedure, a variety of substance-related criteria have to be considered, for instance, the potential for persistence in the environment, the bioaccumulation potential within different organisms, endocrine disrupting properties or the toxicity of a chemical substance. Further criteria also influence the outcome of the final assessment, e.g., the chemical’s relevance for regulation or the exposure of the substance to consumers or the environment. In summary, substance assessment is a knowledge-intensive and time-consuming process that requires high levels of different domain expertise. To concentrate on those substances of highest regulatory priority usually a screening of substances is conducted in order to filter-out substances without harmful properties. The screening process, however, also requires domain expertise and manual efforts.

Facing the challenges stated above the German Environment Agency initiated a strategic project to improve the effectiveness and efficiency of the substance screening and assessment process. In initial requirements analysis workshops all participating stakeholders (experts, management, IT) were interviewed. The desired goals, current bottlenecks, and future chances were identified and aligned with the team. The ultimate goal of the project was the implementation of collaborative decision support and documentation software having the following desired advantages:

- Definition and implementation of standardized decision processes.
- Centralized documentation and casebook of substances.
- Centralized documentation of work plans and decisions.
- Collaborative work of different teams on the same substance at the same time.
- Automated data processing on large amounts of data (especially for screening tasks).
- Quick distribution of new knowledge (short familiarization and training periods).
- Availability of a knowledge archive (e.g. when colleagues leave).

This paper reports on the web-based collaborative wiki system KnowSEC (“Managing Knowledge of Substances of Ecological Concern”) that was developed to fulfill the requirements from above. For automated and standardized decision support we integrated semantic technologies and strong problem solving methods into the tool. Furthermore, we implemented methods and process models to enable collaboration between the team users. We share our experiences with the implementation and use of the system.

The rest of the paper is organized as follows: The methods used for semantic representation, knowledge-based decision support, and collaboration are described in “Methods” section. The results about the design concept on the knowledge level and the implemented system are given in “Results” section. The paper is concluded in “Conclusions” section with a summary of the presented work, a discussion of related work and an outlook of future developments.

## Methods

From the functional requirements stated above we derive the following technical requirements, for that we need to identify appropriate methods:

- R1: Flexible representation of data and knowledge, in order to easily include new types of knowledge.
- R2: Expressive knowledge representation for implementing decision processes on substance screening and evaluation.
- R3: Tools for centralized documentation and presentation of knowledge.
- R4: Tools for collaborative decision work.

The first two items are related to knowledge representation issues, whereas the last two items deal with technologies for collaboration in knowledge management. We discuss appropriate methods for these items in the following sections.

### Knowledge representation

**Fig. 1:**
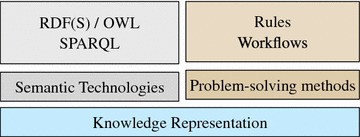
The stack of discussed knowledge representation methods

As depicted in Fig. 1, we combine semantic technologies [1] and strong problem solving methods [2, 3] into a coherent approach. Whereas semantic technologies allow for a flexible representation and access to knowledge and data, we also include strong problem solving methods to implement expressive decision knowledge. At its core, the system needs to represent all work processes and collected data around chemical substances.

**Fig. 2:**
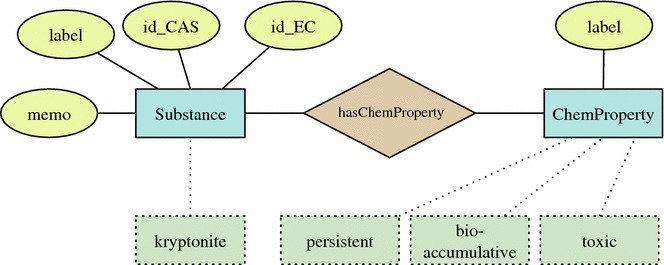
A graph visualization representing the concepts Substance and ChemProperty together with their primitive properties (connected by *solid lines*) and the class property hasChemProperty. A concrete example is given in the *dotted boxes*

That way, a substance-centric work on chemicals groups all knowledge and actions around the particular substances. For example, the system represents known identifiers of substances, the already known properties of substances, and all members currently working/have worked on the substances. Figure 2 depicts a simplified graph visualization modelling the knowledge about a specific substance. Solid lines depict primitive property relations for the classes *Substance* and *ChemProperty*. For instance, the identities EC and CAS number, a textual memo, and a name label are connected with the class *Substance*. Also the class *ChemProperty* has a name label. The class relation *hasChemProperty* connects classes by a diamond. Example instances are given below in dotted lines: For demonstration purposes, we use as example the fantasy substance *Kryptonite*, for which we define persistent, bioaccumulative, and toxic properties.

**Fig. 3:**
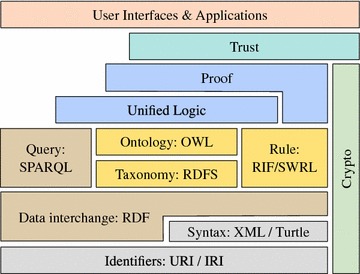
The stack of semantic technologies

Traditionally, such knowledge was stored in relational or XML-based databases [4, 5]. A more expressive and flexible approach is provided by ontology languages, that were standardized in the context of the Semantic Web initiative [1]. The World Wide Web Consortium (W3C) recommended a number of standards building the layered stack of semantic technologies (see Fig. 3) for representing and persisting relational and production knowledge, for querying the included data and for reasoning over the knowledge. The stack is based on fundamental web technologies such as URI/IRI and XML, where URI/IRI are standards for defining unique locations for resources, such as web pages or web-accessible things. XML [6] is a standard for representing and accessing structured data. Most prominently the Resource Description Framework (RDF) defines a standardized language for connecting arbitrary knowledge resources with relational properties [7].

The Resource Description Framework Schema (RDFS) builds on RDF and introduces a standardized data model with the possibility to define (a hierarchy of) classes with a corresponding hierarchy of properties [8]. The Web Ontology Language (OWL) refines RDFS by expressive property constructs (e.g., transitive, disjoint, inverse) and classes (e.g., complements, unions, and closed classes) [9]. Ontologies are defined in RDF(S) or OWL and the SPARQL (SPARQL Protocol And RDF Query Language) language [10] is used to query and update ontologies. The remaining technologies of the semantic web stack are not further touched in the context of this paper.

A simple example briefly demonstrates the modelling and querying with ontology languages. The entities shown in Fig. 2 can be modelled in OWL. Substances and chemical properties are implemented as the OWL classes ks:Substance and ks:ChemProperty, respectively. Further, we define simple Datatype properties to connect string attributes to the classes. A number of chemical properties is defined as instances of ks:ChemProperty: persistent, bioaccumulative and toxic. The Turtle notation [11] is used for the following listings.
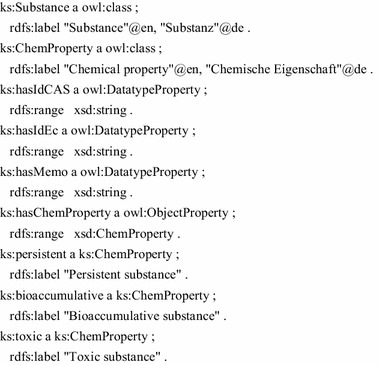


A special class of substances is defined by PBT substances, i.e., chemical substances having the persistent, bioaccumulative, and toxic property. Such a substance type can be easily defined as the complex OWL class ks:PBTSubstance.
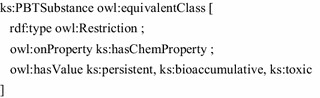


Based on that simple model, the data of the concrete chemical substance Kryptonite is then added as an RDF instance.
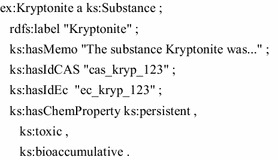


Practically, the ontology is located in a triple store and the elements can be accessed by query/programming interfaces [12–14]. The following SPARQL query simply lists all PBT substances and their labels.
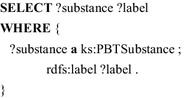


Also the non-existence of relations can be queried with SPARQL. That way, the following query lists all substances, that are not classified as PBT substance.
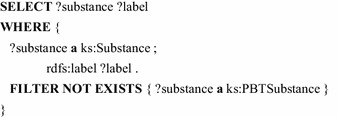


RDF(S), OWL, and SPARQL provide flexible methods to represent and query properties of chemical substances. However, the actual derivation of a chemical property, such as persistence or specific types of toxicity, is a complex and knowledge-intensive task. The inference of the property is then based on a complex decision process, where often partial sub-decisions are aggregated into a final decision whether to derive a specific property [15].

*Strong problem solving methods* offer the appropriate technology to represent and process such complex decision knowledge. Those methods origin from classical expert systems [2, 16] by implementing human expert knowledge in software systems. Typical classes of such systems are recommender systems, planning and configuration tools, and classification systems. A decision support system uses classification mechanisms to propose appropriate decisions for a given problem statement. For instance, with the attribute data about a chemical substance the system derives assessment properties such as the persistence, bioaccumulation, and toxicity. In the past, knowledge-based decision support systems were successfully deployed in different domains; see for instance [17–23]. Over the years a wide range of knowledge representations [24] with different characteristics was developed. The classic and most generic knowledge representation are rules [25–28]: They provide a flexible notion for deriving instances of the ontology. With (RIF) Rule Interchange Format [29] a standardized rule language is already defined. The following rule is written in a more generic notion and shows the derivation of a *negative biodegradation property* when at least one of three different thresholds is not reached. Here, the ECHA abbreviations are used for the properties “theoretical CO_2_ evolution” (ThCO2), “theoretical oxygen demand” (ThOD), and “dissolved organic carbon” (DOC), respectively.
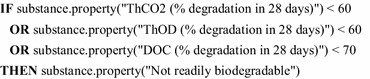


A more process-oriented view of the knowledge is implemented by decision trees [30], business process models [31], or diagnostic workflows [32].

**Fig. 4:**
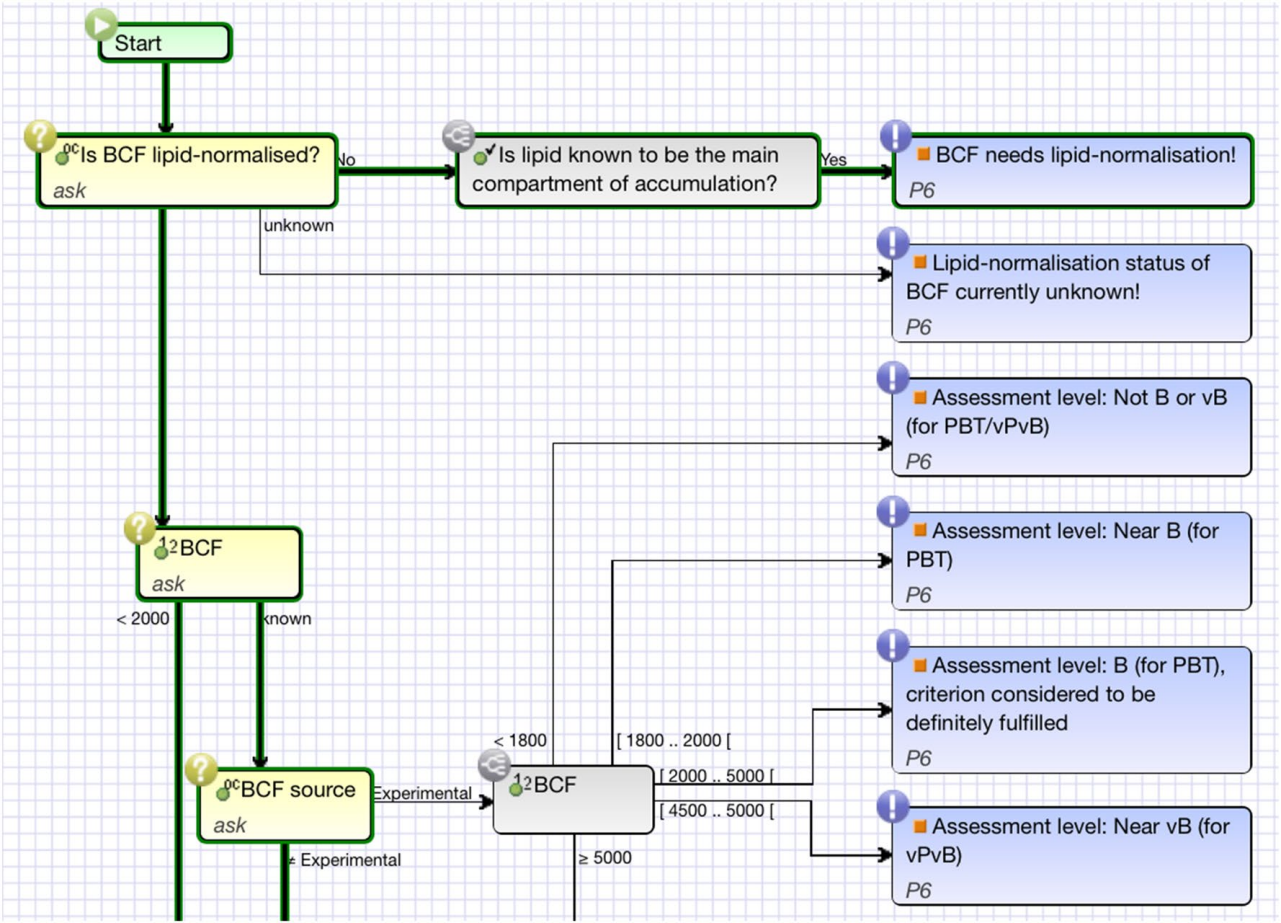
A simplified workflow for assessing bioaccumulation-related decisions. The derivation of the decisions concerning B (bioaccumulative), vB (very bioaccumulative), PBT (persistent, bioaccumulative, atoxic), and vPvB (very persistent and very bioaccumulative) are also based on the value and source of the bioconcentration factor (BCF). For a particular substance the reasoning trace is highlighted by *bold green lines*

Figure 4 depicts an excerpt of a simplified version of a bioaccumulation potential assessment. Here, the relevant questions are asked in a decision tree-like structure. On the right side of the figure, corresponding properties of the substance are derived. For a concrete substance the reasoning trace can be displayed in bold green. The trace is useful for explaining particular decision results.

The developed approach KnowSEC combines the aforementioned semantic technologies and strong problem solving methods in a coherent manner, as we will see in “Results” section.

### Collaboration

**Fig. 5:**
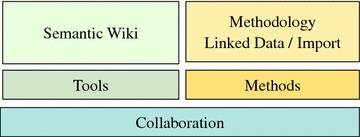
Stack of collaboration methods

From the requirements in “Background” section we derived the need for collaborative knowledge management technologies. The work on substances denotes a collaborative process of participants with different domain expertise. The results of the process and the knowledge about the current decision process need to be presented and edited in a centralized manner. As depicted in Fig. 5 we discuss collaborative tools enabling the distributed engineering of knowledge bases and we describe appropriate engineering methods supporting the collaborative work.

**Fig. 6:**
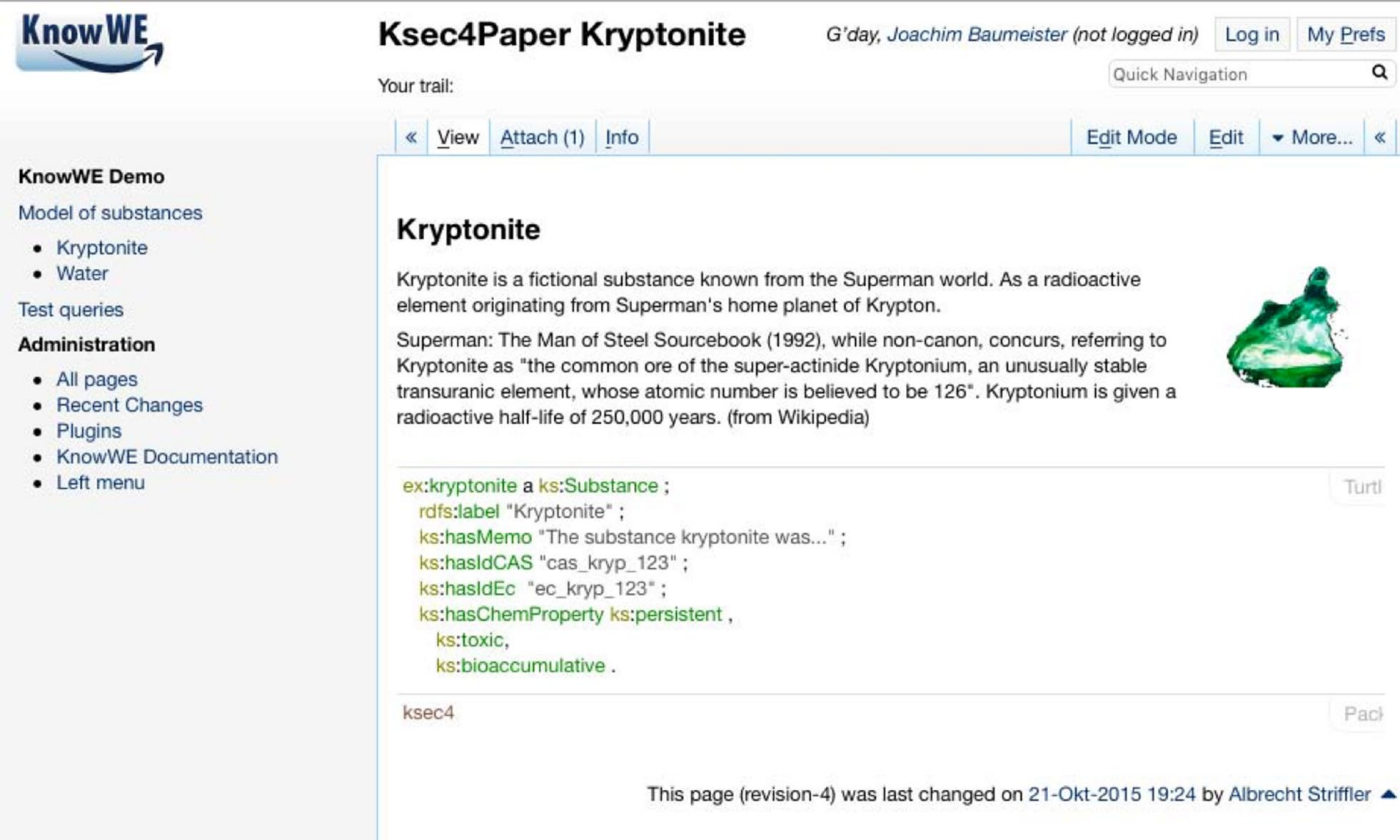
The example ontology edited in the semantic wiki KnowWE

There exists a number of software tools supporting the collaborative knowledge engineering and decision making. Today, web-based environments for the collaborative engineering of ontologies exist, for instance, WebProtégé [33, 34] and WebODE [35]. WebProtégé is a typical example of such tools, where classes and instances are created and maintained by using dedicated forms and graphical editors. Collaboration is supported by discussion panels about specific engineering decisions and version control management, as discussed in [36, 37]. In recent years, wiki systems proved to be an alternative easy-to-use collaborative system approach with a higher level of flexibility. The content of the wiki is a collection of interlinked web pages, i.e., the wiki articles. The articles are not only viewed, but also directly edited within a standard browser. For the text formatting and media inclusion a simplified markup language is used. As an extension, *semantic wikis* [38] add further markup to edit and maintain semantic knowledge bases within the wiki articles. The knowledge base can be freely distributed over the pages of the wiki, as for instance implemented by Semantic MediaWiki [39] and KnowWE [40]. Figure 6 shows an article in the semantic wiki KnowWE with a part of the example ontology from above. We see that ontological definitions—here the Turtle definition of the substance Kryptonite—is placed in the article and can be mixed with text and images.

Besides the application of appropriate tools, successful collaboration also depends on the use of collaboration methods. Like in classical software engineering the use of best practices and development conventions improve the joint work. In knowledge engineering, different approaches investigate this issue [41, 42, 43].

Typically, collaboration is defined as the joint work between humans pursuing a specific goal. We additionally see the (re)use of decisions and data collected by external agents as a collaborative task. The possibility of linking data and knowledge of arbitrary sources was one of the major goals of the semantic web initiative. The term Linked Data [44, 45] refers to the representation of ontologies, so that single data items can be accessed by technologies such as RDF and SPARQL. Due to the standardized access of data items, the inter-linkage becomes practically possible. The approach of linked data has been successful with respect to the continuous growth of the Linked Open Data cloud, as regularly analysed [46]. In (closed) enterprise systems, linked data also plays an increasing role since the publication of knowledge and data can be standardized and based on existing implementations. For older information systems, typically specific import interfaces are provided to connect included information into the linked data cloud.

## Results

We developed the decision support and documentation system KnowSEC (“Managing Knowledge of Substances of Ecological Concern”). Since 2012 the system is internally used by the section *Chemicals* of the German Environment Agency (Umweltbundesamt) and underwent regular improvement updates. KnowSEC builds on the open-source wiki system KnowWE (http://www.d3web.de) and is extended by plugins tailoring the collaboration support during decision making on chemical substances. All information is represented by linked ontologies.

### Substance-centric system design

**Fig. 7:**
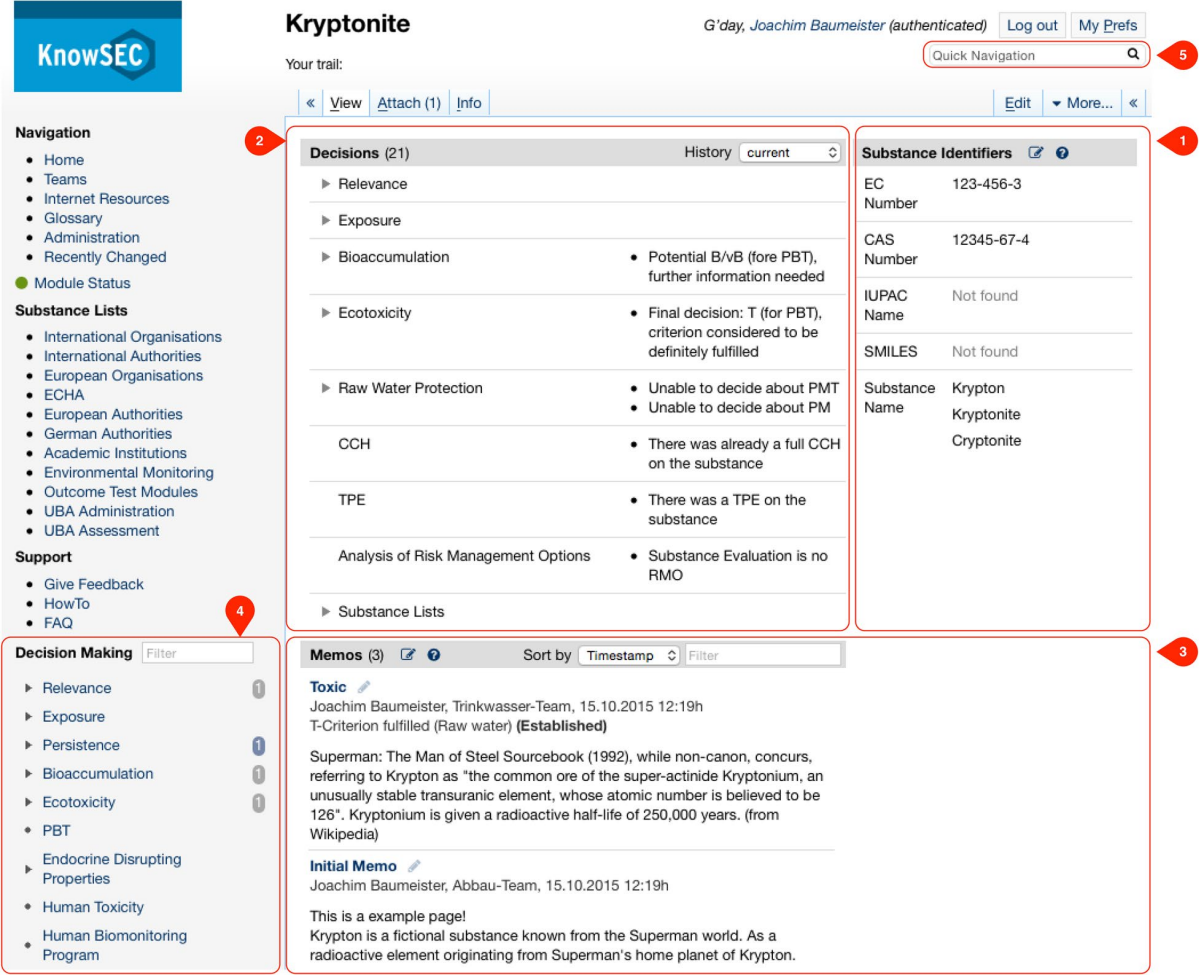
The article of the substance Kryptonite showing the known identifiers (*1*), the summary of known decisions (*2*), the informal memos written for the substance (*3*), a link to the automated decision interviews (*4*), and a search slot for accessing substances (*5*)

The *chemical substance* is the unifying mental concept for the organization of information in KnowSEC. That way, decisions and documents usually correspond either to a single substance or a group of substances. The system is designed, so that each substance is represented by a distinct article in the semantic wiki. Every substance article presents the relevant information about the particular substance. Figure 7 depicts the substance article of the example substance *Kryptonite*. We introduce the shown panels of the article in the following.

#### Identifiers

For a new registration, a new substance article is created listing the known identifiers EC number, CAS number, IUPAC name, SMILES code, and internal naming. Authorized users are able to directly add or clarify identifiers within the wiki article (see edit button in Fig. 7-1).

Substances can be accessed by their known identifiers by using the semantic search slot (Fig. 7-5). Especially in the early phases of the assessment the identification and the labelling of substances new to the system is very important in order to group duplicate registrations or to label registrations properly. The ontological representation of identifiers even allows for the definition of competitive/contraindicative identifiers. Predefined SPARQL queries, however, report such inconsistencies through a continuous quality dashboard of the system [47]. There also exist declarative queries, that check entered identifiers with respect to known checksums or duplication. Authorized team members are also able to define their own SPARQL tests within the running system for application-specific quality checks. That way, the identification process can be collaboratively performed. The information about the provenance of all changes is available to the users, i.e., the changed identifier, the date of change, and the acting team member is transparent for all users.

#### Decisions

**Fig. 8:**
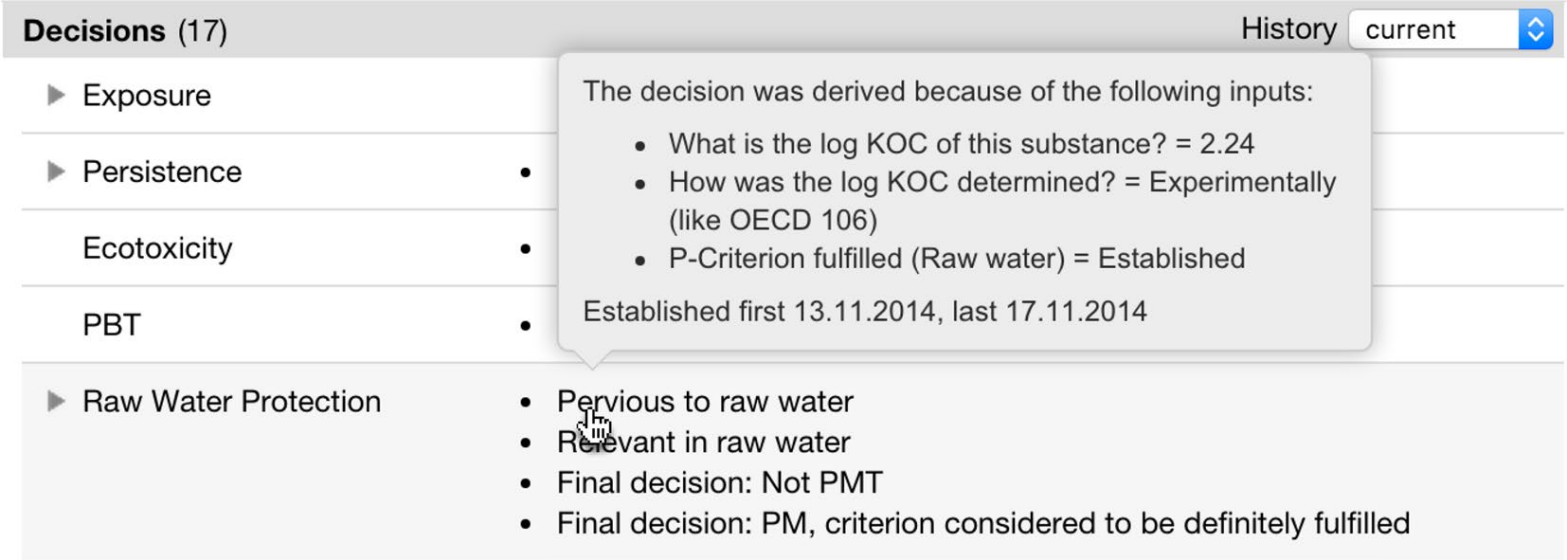
The explanation for a selected decision shows the responsible facts with corresponding entry date

Currently valid decisions concerning the presented substance are listed prominently at the top of the article (Fig. 7-2). The derived decisions are grouped according to the decision modules available in the system and the final decisions are shown at first glance. On demand, partial decisions are unfolded, which often provides valuable detail information about the current state of the assessment of the substance. Previously valid decisions can be viewed through a time-machine available with the menu *History* in the decision panel. In case of an unclear decision, KnowSEC is able to display an explanation for a made decision: When hovering over a decision name, the responsible facts with date are shown (see Fig. 8). Analogously, facts influencing the inference can be explained to the user. Also, the reasoning trace of workflow models can be highlighted in order to describe the decision process to the user.

#### Documentation

Often, users add specific information about a substance, for example describing the justification for a concrete decision. We call this type of information *decision memo* (Fig. 7-3). The memos considering the selected substance are listed in the lower part of the article. They can be directly created and edited within the article. Also, a formal decision can be added to a memo; for instance in Fig. 7-3 the decision *T*-*Criterion fulfilled (Raw water)* is attached to the memo with the title *Toxic*.

All memo information including the provenance data is represented in the ontology for further use such as semantic navigation and filtering. Besides memos, the system can also draw formal decisions on a substance using the *decision making* panel (Fig. 7-4). The panel shows substance criteria for which automated decision modules were implemented, for example for the assessment of the persistence, bioaccumulation and toxicity of the selected substance.

**Fig. 9:**
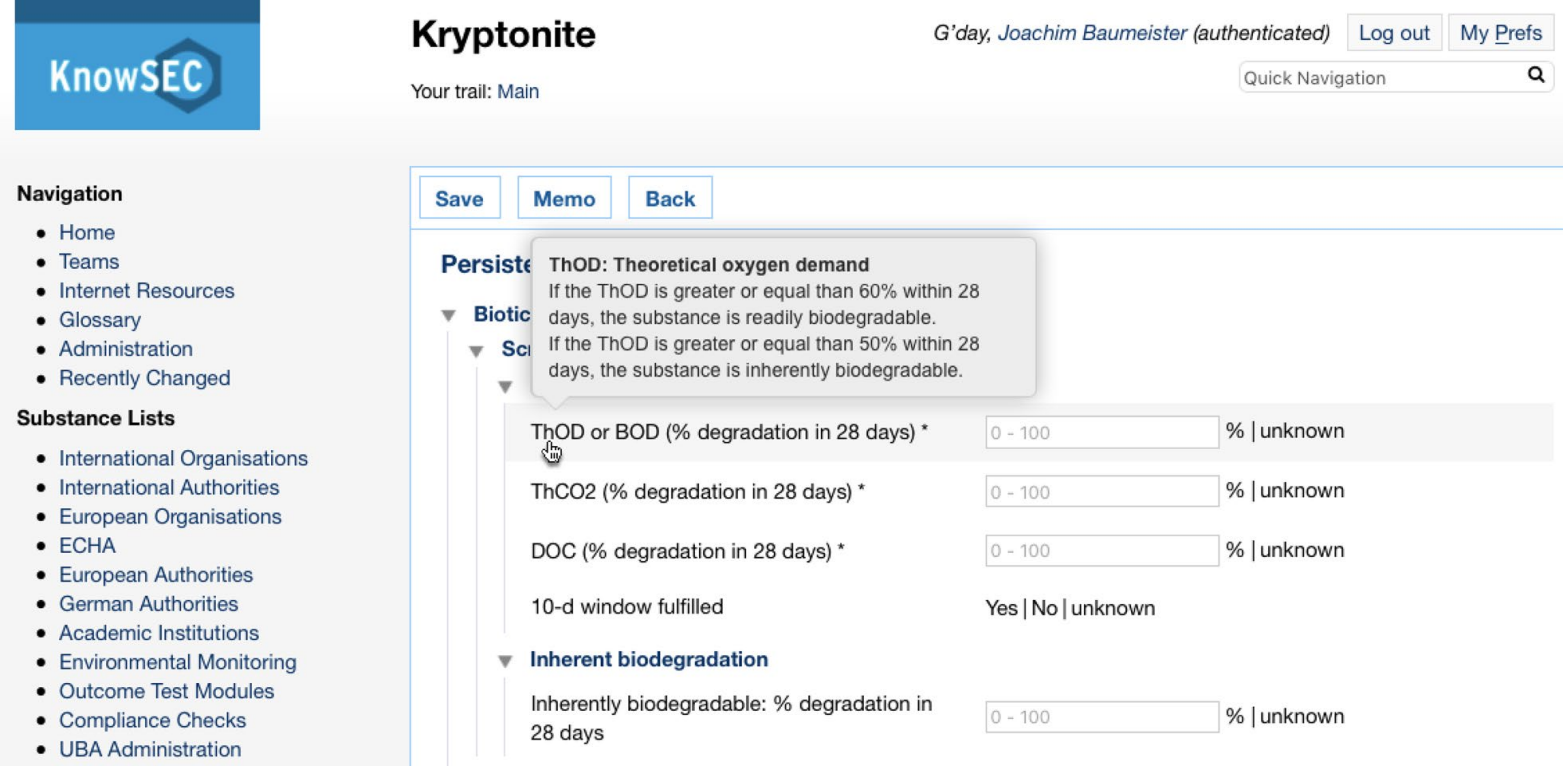
Interactive interview for decision making about the biotic degradation properties (persistence) of the fantasy substance Kryptonite. On demand, an explanation text is displayed for the selected question

When clicking on such a module the system starts an interactive interview with the user, where properties/data about the substance are asked in order to conclude a decision about the chosen criteria; see Fig. 9 for an example interview concerning the persistence of a substance. Here, strong problem solving methods were used to implement expert system-like behaviour.

### Semi-automated decision making

**Fig. 10:**
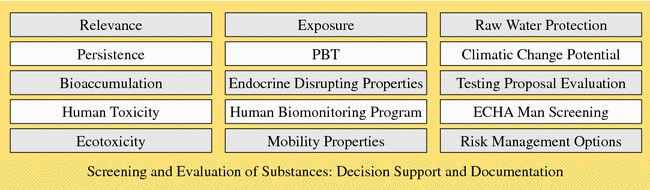
Decision modules of the system KnowSEC

KnowSEC provides interactive decision support for the user. When clicking on a criterion in the decision making panel (Fig. 7-4) the corresponding interactive interview is started. One design goal of KnowSEC was the availability of decision modules for the most relevant criteria that are required to assess a substance appropriately. Figure 10 depicts the modules that were developed within KnowSEC over the past years.

For each criterion, a hierarchy of decisions is defined that organizes the possible outputs of the module. Each criterion module corresponds to a knowledge base that can be developed directly in the wiki system. The used knowledge representation varies and depends on the internal complexity of the domain knowledge. For example, the module for the criterion *Persistence* was mainly implemented using non-monotonic scoring rules [48]. The assessment of *Bioaccumulation* was implemented using workflow knowledge (see Fig. 4). All decisions and questions asked by the particular modules are connected by the ontology. That way, the question and its value of one module can be reused in another module. Also, the derived decisions of a module can be reused by another module. For instance, the module *PBT* uses the decisions of the modules *Persistence, Bioaccumulation*, and the different toxicity modules to derive an assessment about the state “PBT”, which is a central criterion for the identification of an SVHC (substance of very high concern) for the environment.

**Fig. 11:**
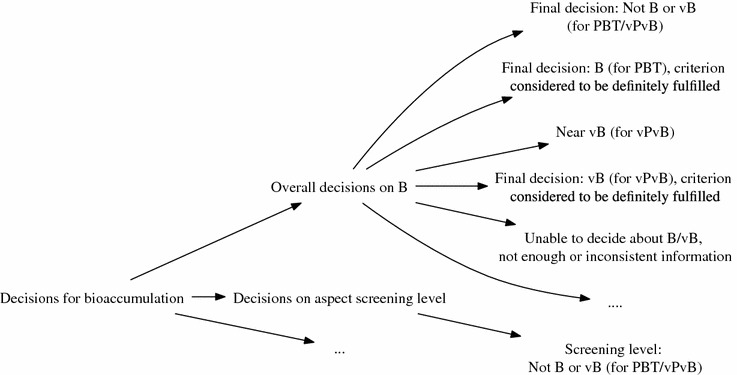
Part of the hierarchy to represent the possible decisions concerning the bioaccumulation (B) of a substance, including the classes vB (very bioaccumulative), vPvB (very persistent and very bioaccumulative), and PBT (persistent, bioaccumulative, toxic)

Currently, the ontology connects 916 hierarchically structured decisions (see Fig. 11 for an excerpt) and 393 hierarchically structured input questions. The value type of the questions can be multiple/one-choice values, numeric inputs, date values, and text values.

The decision modules of KnowSEC deliver a significant added-value when compared to standard substance databases. However, besides the assessment of substances the semi-automated decision modules also support the steering and monitoring of the decision process itself. Here, data about the decision process of the substance itself is documented. That way, the current assessment of a substance is represented in the ontology. Specialized decision modules collect the status data and provide guidance for the next steps in a substance’s workflow. For instance, there exist interactive modules for documenting and monitoring the need and outcome of certain REACH procedures like for example the commenting of so-called compliance checks or the evaluation of testing proposals. Aggregated views on the substance status and lists of substances being in a specific status (or combination of status) can be queried and listed in the system. The next section describes the dynamic view feature of KnowSEC in more detail.

### Dynamic views on the decision process

Dynamic views were introduced in KnowSEC in order improve the overview in the collaborative setting. Such views query a specific state of the decision process. They can be inserted into any wiki article and are updated on every article visit (thus “dynamic”).

KnowSEC implements dynamic views with SPARQL queries that are embedded into the article. When presenting the article in the view mode the result of the query is shown as a table or visualization; KnowSEC provides a number of different visualizations for SPARQL queries. Since, any information—ranging from substance identifiers and informal memos to formal decisions—is represented as ontological descriptions, SPARQL can reach almost any known information state of the system. The following query lists all substances that are currently screened by the agency as PBT substances (persistent, bioaccumulative and toxic). Please note the different linking to chemical properties compared to the simplified example in Section “Knowledge representation“.
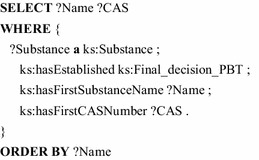


The simple definition of dynamic views yielded a collection of tailored overview pages that are directly linked in KnowSEC (see header “Substance Lists” in the left panel of Fig. 7). Custom views on the data support the team-work on the substances in a significant manner, since the specialized views help to organize the different aspects of the substance work. Every view can be exported as a MS-Excel sheet for external use.

### Knowledge and information harvesting

The knowledge is usually generated and provided by the experts at the German Environment Agency. The substance identities mainly refer to substances registered under REACH, additional information and substances are provided by experts on substance identity working at the agency. As KnowSEC is no standard substance information database but a knowledge system, the use of chemical properties in the form of measured values is mostly avoided. The decisions of experts are mainly used on them. These follow the standards set out within the respective guidelines of the REACH regulation and the internal provisions used by the agency. When it is necessary to use measured values, these are most often taken from REACH registration dossiers or relevant scientific reports. As with all measured data used within REACH the Klimisch score [49] is used to assign the reliability.

### Application und use cases of the system

The KnowSEC system is mainly used in three general areas during the assessment of chemical substances: The documentation of decisions (automatically or manually derived), the provisioning of substance overviews and their corresponding states, and for decision support during the assessment.

*Documentation* of the entire assessment of a chemical substance: The teams mainly document the screening of substances and the reduction of new substances to a set of “bandits”, i.e., substances that have properties and/or uses, that might necessitate further regulation. The process and the outcomes of the further assessment steps as well as regulatory initiatives are also documented within the system.

**Fig. 12:**
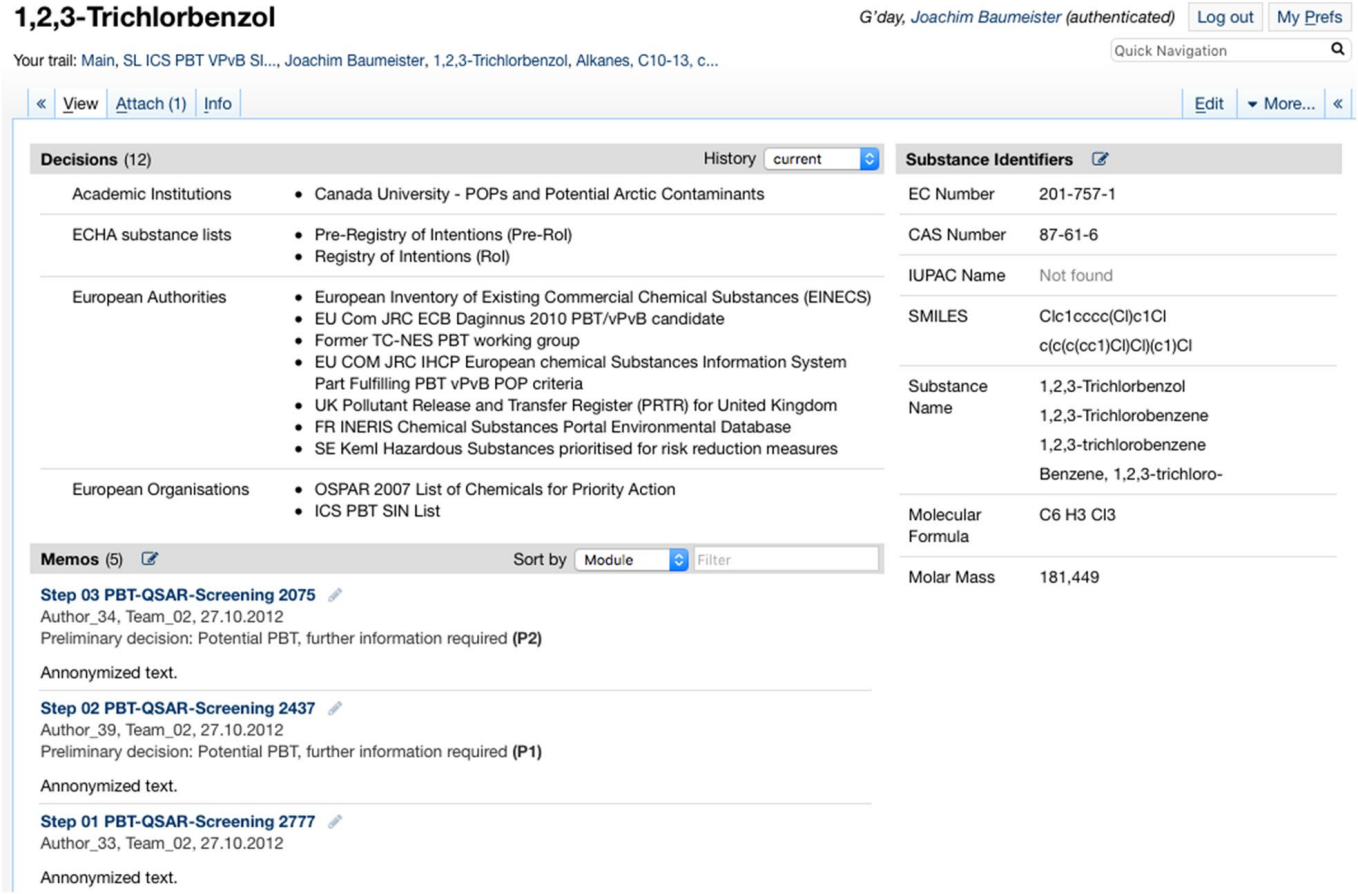
The substance info page of 1,2,3-trichlorbenzol showing the currently derived decisions and the documentation of the assessment done so far

For instance, in Fig. 12 the info page of the substance “1,2,3-Trichlorbenzol” is depicted. The list of memos shows the documentation of a QSAR Screening identifying the substance as a possible PBT substance in steps 01, 02, and 03.

*Provision of overviews* Each substance has an info page providing a comprehensive summary of all data and knowledge collected for the substance. The substance info page collects all information available in the wiki (and probably from connected external sources). The substance info page is seen as a very helpful research entry for a first substance assessment, since also information from previous or parallel work inside and outside of the UBA is displayed on the page.

**Fig. 13:**
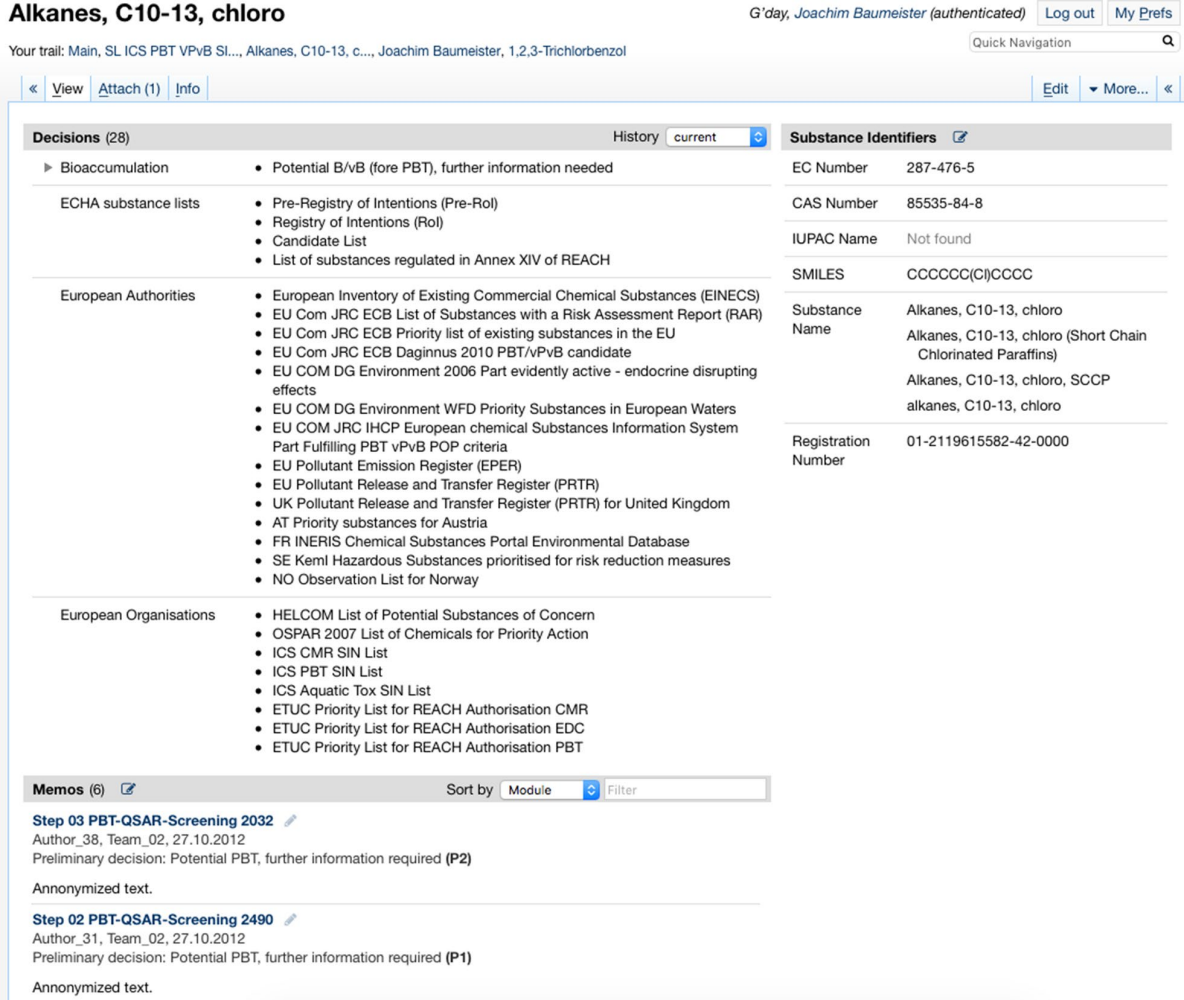
The substance info page of “Alkanes, C10-13, chloro”, summarizing the currently derived decisions, a selection of memos edited and the known substance identifiers

In Fig. 13, we see the overview page of the substance “Alkanes, C10-13, chloro”. Here, the currently derived decisions are shown following by a list of memos edited for this substance. The identifier block on the page is especially helpful for the initial research, since a single substance name can have a different substance identities used in different contexts.

**Fig. 14:**
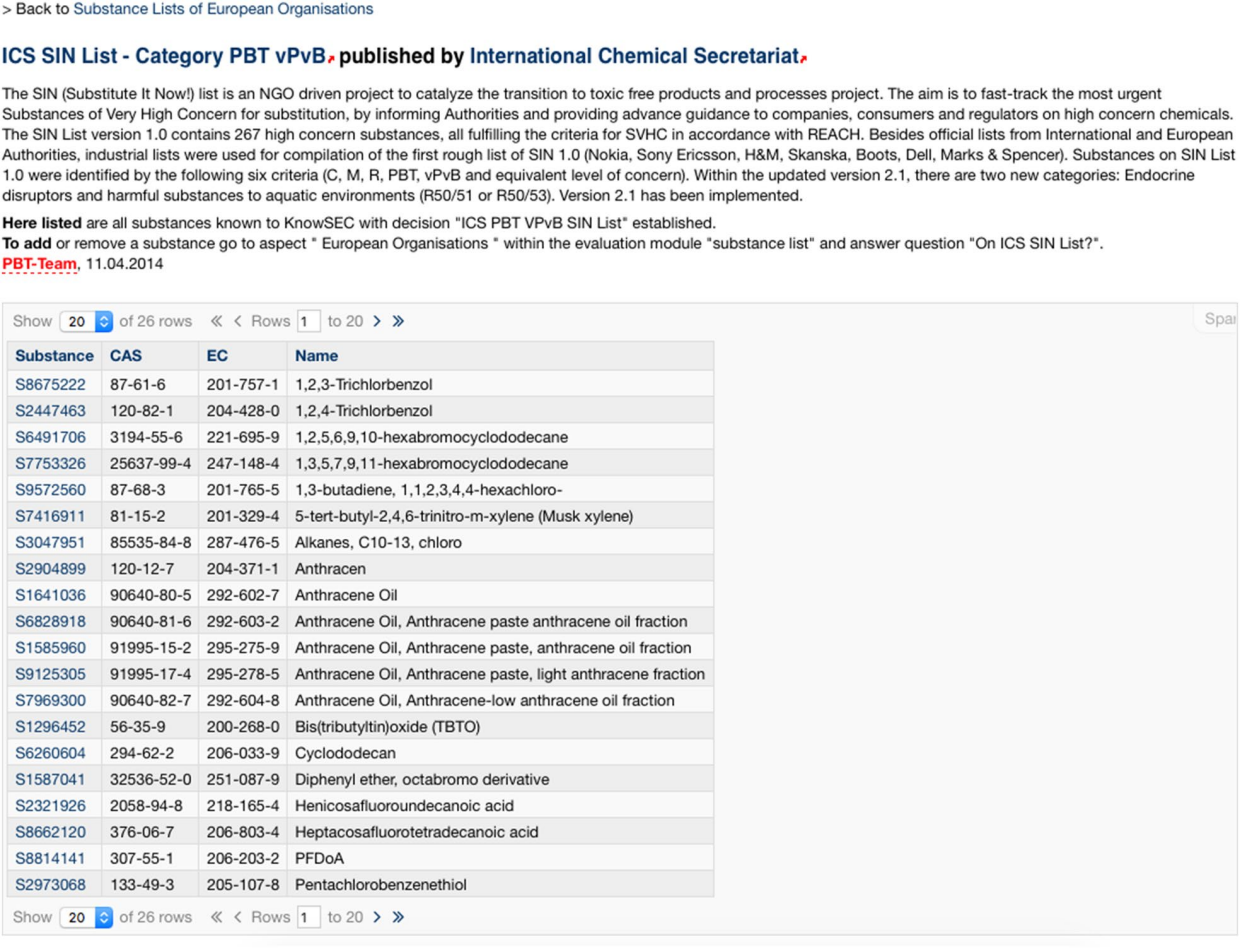
Excerpt of the European ICS SIN List with PBT properties; automatically generated based on a derived decision PBT

Aggregating overviews are automatically generated by SPARQL inclusions, mostly depending on the derivation of selected decisions. In Fig. 14, an excerpt of PBT substances of the European ICS SIN List are automatically listed. Changes made to the decisions belonging to the substances in the system are transparently propagated to these overview lists.

*Decision support* The KnowSEC system provides a comprehensive collection of decision modules supporting the user during the substance assessment. In Fig. 9, an excerpt of the PBT assessment is shown. Especially for non-domain experts or assessors new to a specific assessment domain, the feature of getting quality controlled support from automated modules is useful: On the one hand, entered data automatically provides the results for standard assessment on the substance info page. On the other hand, the decision module can be used as a kind of “check list” for questions to be considered during an assessment.

## Conclusions

### Summary

The work on chemical substances under the REACH regulation is a complex task that requires the collaboration between subject matter experts. We introduced the knowledge-based decision support and documentation system KnowSEC that was built to support this task. Since 2012 KnowSEC is in regular use by the unit *Chemicals* of the German Environment Agency (Umweltbundesamt). In the following, we discuss the contributions of the system with respect to the requirements stated in the beginning of the paper.

#### Ontologies and strong problem solving methods

Standardized ontology languages (RDF/OWL and SPARQL) in combination with strong problem solving methods (scoring rules, decision trees, workflows) were the key enabler for an effective and flexible representation of knowledge. A large semantic graph with more than 6,000,000 statements covers all data about the included substances, whereas decision modules add knowledge about derived substance criteria into the graph. Over the years of use, the representation of substances was extended and slightly refactored multiple times by edits of the ontology model. Also, the decision modules were extended and adapted by adding new rules or workflow knowledge. Continuous integration techniques implemented in KnowSEC helped to automatically monitor the quality of the knowledge base. The use of a semantic wiki allowed to implement all these changes during runtime and thus proved to be an effective web-based platform, where the knowledge bases and the substance data can be easily retrieved and curated. In summary, requirement R1 (flexible knowledge representation) and requirement R2 (expressive knowledge representation) are fulfilled.

#### Centralized documentation and presentation of knowledge

The organization of the information through *substance articles* provides an intuitive view on substances. All information about a specific substance is aggregated on the designated substance, especially its derived decisions, the written memos, and the known identifiers. The semantic search interface (Fig. 7-5) and the dynamic views provide an efficient access to the substances of the system. As a web-based system KnowSEC only requires a web-browser for end-users. In summary, requirement R3 is fulfilled by the current implementation of KnowSEC.

#### Successful collaboration on knowledge and substances

The engineering of the knowledge-based modules started in 2011 with a small group of three subject matter experts, that increased to eleven experts in total over the years. The experts are organized in domain-specific teams, so that each team works on the designated aspect of their own expertise, e.g., persistence in the environment or bioaccumulation potential.

Following agreed knowledge engineering conventions, the modules are structured in a comparable manner, and terms of the knowledge base follow naming conventions; decisions are structured similarly. The ongoing development of new modules was simplified and the maintenance of existing modules, e.g., new partial decisions, was made easier. Internally, the teams are backed by two knowledge engineers that supervise the engineering conventions and that are responsible for initiating small refactorings/reorganizations of the total knowledge base.

At the moment, about 13,400 substances are represented in KnowSEC—more substances will be included with the upcoming registration phase of the EU REACH regulation in 2018. A high number of substances and corresponding substance data were imported into the system by using the provided standard MS-Excel interface of KnowSEC. That way, prepared MS-Excel sheets helped to quickly elicit information about many substances. In consequence, parts of the automated decision modules were used to filter relevant substances in a series of screening phases. Here, dynamic views supported the management of the screening phases. As of 2015 the current installation of KnowSEC includes articles for about 13,400 substances having about 76,000 documented (sub-)decisions. In summary, requirement R4 (tools for collaborative decision work) is fulfilled by the current implementation of KnowSEC.

### Related work

A design approach related to KnowSEC is described by the web-based system HERMES [50]. Classical decision making is combined with argumentative discourse among decision makers. The argumentation in the system is comparable to the decision steps decision memos and decision dialogs of KnowSEC. However, HERMES provides an elaborated approach for the discussion and argumentation before making the actual decision. Collaborative decision making is also discussed by Palomares et al. [51], where multiple experts are supported to make a unified decision in a management-based approach. The tool MENTOR is described, where the multiple opinions of the contributors and the evolution of their opinions is visualized. KnowSEC supports collaborative decision making by explanation interfaces that are generated by declarative SPARQL queries. The Maritime Integrated Decision Support Information System on Transport of Chemical Substances (MIDSIS-TROCS) provides a data base on substances spilled at sea [52]. MIDSIS-TROCS contains a decision support tool that decides about behaviour classification for chemical substances. The scope of the decision modules in KnowSEC covers a broader range of questions, and the knowledge representation of MIDSIS-TROC is limited to decision trees. Also active users cannot add new decision knowledge to the running system; this holds for tacit decisions added by decision memos but also structured substance data. In [53] the decision support system Bioclipse-DS is introduced for supporting the primarily chemical liability assessment. In the shape of an expert system it is focused on the special task of liability assessment. It combines similarity searches, structural alerts and QSAR models. Like KnowSEC the expert knowledge can be also extended by a plugin mechanism. KnowSEC, however, covers a broader (and shallower) range of chemical assessment, but in contrast to Bioclipse-DS offers strong support for the collaboration and documentation of the assessment work. Verdonck et al. [54] introduce a knowledge-based system for supporting the screening of substances registered under REACH. Following a conservative approach, substances with very low or no immediate concern are filtered-out. Here, also key environmental parameters are used for the derivation. KnowSEC also include screening components for some substance criteria (persistence, bioaccumulation, toxicity), but also emphasizes the documentation of the screening process.

### Future work

At the moment, most data items are stored statically in KnowSEC. Decisions on the substances are either derived using that data or are provided by decision memos. From the beginning, KnowSEC was designed as an interactive decision support and documentation system but not as a substance data base. That way, the linkage to external substance data needs to be emphasized more in the future. With the advent of semantic-aware information communication technologies the connection to external chemical databases becomes feasible. That way, the semantic linkage can dynamically integrate generic information about particular substances, such as physical/chemical data, without the need for local storage. In summary, a *linked chemical data cloud* can emerge and can be used for a variety of advanced chemical services.

The system is currently used only by one chemical agency. We see potential for a shared effort within the European Union, where other agencies working under REACH are required to implement the same substance assessment procedures. Here, the potential for exchange between the agencies at the knowledge level clearly exists, also from a technical perspective. The current approach then needs a further refinement concerning the management of authorization roles for substance data in order to keep the internal processes of agencies internal.

## Availability and requirements

Project name: KnowSEC

Demo available: http://knowsec-demo.denkbares.com

Project home page: http://www.d3web.de/

Operating system: Platform independent

Programming language: Java

Other requirements: Java 8 or better, Apache Tomcat 6.0 or better

License: GNU LGPL 3.0

## Abbreviations

CAS: Chemicals Abstracts Service
ECHA: European Chemicals Agency
IUPAC: International Union of Pure and Applied Chemistry
KnowSEC: System: Managing Knowledge of Substances of Ecological Concern
KnowWE: System: Knowledge Wiki Environment
OWL: Web Ontology Language
PBT: Persistent Bioaccumulative Toxic
RDF: Resource Description Framework
RDFS: Resource Description Framework Schema
REACH: Registration, Evaluation, Authorisation And Restriction Of Chemicals
RIF: Rule Interchange Format
SMILES: Simplified Molecular Input Line Entry Specification
SPARQL: SPARQL Protocol and Resource Query Language
UBA: Umweltbundesamt
URI/URL: Unified Resource Identifier/Unified Resource Locator
W3C: World Wide Web Consortium
XML: Extensible Markup Language
